# Supplementary material for: Mechanism of SK2 channel gating and its modulation by the bee toxin apamin and small molecules
Source: eLife. 2025 Dec 4;14:RP107733. doi: 10.7554/eLife.107733 (PMC12677897; doi:10.7554/eLife.107733)
Supplement: Supplementary file 1. [file elife-107733-supp1.docx]

**Data collection parameters and Refinement statistics**

| **Structure** | **SK2-4/CaM + Ca^2+^** | **SK2-4/CaM Ca^2+^-free** | **SK2-4/CaM + apamin** | **SK2-4/CaM + compound 1** | **SK2-4/CaM + compound 4** |  |  |  |  |
| --- | --- | --- | --- | --- | --- | --- | --- | --- | --- |
| PDB | 9O48 | 9O51 | 9O52 | 9O53 | 9O5O |  |  |  |  |
| EMDB | EMD-70089 | EMD-70120 | EMD-70121 | EMD-70122 | EMD-70145 |  |  |  |  |
| **Data Collection/Processing** |  |  |  |  |  |  |  |  |  |
| Microscope | Krios | Krios | Krios | Krios | Glacios |  |  |  |  |
| Voltage (kV) | 300 | 300 | 300 | 300 | 200 |  |  |  |  |
| Magnification | 75,000x | 75,000x | 75,000x | 75,000x | 120,000x |  |  |  |  |
| Nominal defocus range (µM) | -0.8 to -1.6 | -0.8 to -1.6 | -0.8 to -1.4 | -0.8 to -1.6 | -0.8 to -2.0 |  |  |  |  |
| Pixel size (Å) | 0.845 | 0.845 | 0.845 | 0.845 | 0.854 |  |  |  |  |
| Total dose (e^-^/Å^2^) | 50 | 50 | 50 | 50 | 35 |  |  |  |  |
| Exposure time (s) | 3 | 3 | 3.05 | 3 | 28 |  |  |  |  |
| Micrograph number | 10,123 | 8,928 | 15,779 | 7,300 | 3,495 |  |  |  |  |
| Symmetry | C4 | C4 | C1 | C4 | C4 |  |  |  |  |
| Initial particle number | ~8.3 million | ~8.2 million | ~7.5 million | ~1.9 million | ~1.6 million |  |  |  |  |
| Final particle number | 658,368 | 386,845 | 734,192 | 262,274 | 346,529 |  |  |  |  |
| Map resolution (Å) | 3.1 | 3.4 | 3.2 | 3.3 | 3.1 |  |  |  |  |
| FSC threshold | 0.143 | 0.143 | 0.143 | 0.143 | 0.143 |  |  |  |  |
| **Refinement** |  |  |  |  |  |  |  |  |  |
| Model Resolution (Å) | 3.1 | 3.5 | 3.2 | 3.4 | 3.2 |  |  |  |  |
| FSC threshold | 0.5 | 0.5 | 0.5 | 0.5 | 0.5 |  |  |  |  |
| Map sharpening B-factor (Å^2^) | 122.9 | 142.3 | 126.2 | 143.6 | 182.5 |  |  |  |  |
| Map composition |  |  |  |  |  |  |  |  |  |
| Non-hydrogen atoms | 16,094 | 12,302 | 16,185 | 15,628 | 15,742 |  |  |  |  |
| Protein residues | 2,044 | 1,596 | 2,056 | 1,992 | 1,988 |  |  |  |  |
| Ligands | 0 | 0 | 1 | 4 | 4 |  |  |  |  |
| B-factors (Å^2^) |  |  |  |  |  |  |  |  |  |
| Protein |  |  |  |  |  |  |  |  |  |
| SK2-4 chains | 46.72 | 64.36 | 65.19 | 30.84 | 67.53 |  |  |  |  |
| CaM chains | 93.73 | 145.43 | 107.20 | 86.32 | 134.41 |  |  |  |  |
| Ligand | N/A | N/A | 84.36 | 11.28 | 48.74 |  |  |  |  |
| R.M.S. deviations |  |  |  |  |  |  |  |  |  |
| Bond length (Å) | 0.002 | 0.002 | 0.003 | 0.004 | 0.003 |  |  |  |  |
| Bond angle (°) | 0.354 | 0.400 | 0.465 | 0.457 | 0.5 |  |  |  |  |
| Validation |  |  |  |  |  |  |  |  |  |
| MolProbity score | 1.11 | 1.05 | 1.06 | 1.19 | 1.34 |  |  |  |  |
| Clashscore | 3.19 | 2.68 | 2.76 | 4.11 | 6.11 |  |  |  |  |
| Rotamers outliers (%) | 0 | 0 | 0 | 0 | 0 |  |  |  |  |
| Ramachandran plot (%) |  |  |  |  |  |  |  |  |  |
| Favored | 98.61 | 98.71 | 98.62 | 98.17 | 98.83 |  |  |  |  |
| Allowed | 1.39 | 1.29 | 1.38 | 1.83 | 1.17 |  |  |  |  |
| Disallowed | 0 | 0 | 0 | 0 | 0 |  |  |  |  |
